# Silicon particles as trojan horses for potential cancer therapy

**DOI:** 10.1186/s12951-014-0035-7

**Published:** 2014-09-16

**Authors:** Roberto Fenollosa, Eduardo Garcia-Rico, Susana Alvarez, Rosana Alvarez, Xiang Yu, Isabel Rodriguez, Susana Carregal-Romero, Carlos Villanueva, Manuel Garcia-Algar, Pilar Rivera-Gil, Angel R de Lera, Wolfgang J Parak, Francisco Meseguer, Ramón A Alvarez-Puebla

**Affiliations:** Centro de Tecnologías Físicas, Unidad Asociada ICMM/CSIC-UPV, Universidad Politécnica de Valencia, Av. Los Naranjos s/n, Valencia, 46022 Spain and Instituto de Ciencia de Materiales de Madrid, CSIC, Madrid, 28049 Spain; Servicio de Oncología, Hospital Universitario Madrid-Torrelodones, Madrid, 28250 Spain; Departamento de Química Orgánica, Universidade de Vigo, Vigo, 36310 Spain; Fachbereich Physik, Philipps Universität Marburg, Marburg, 35037 Germany; Medcomtech SA, C/ Catalunya, 83-85 Viladecans, Barcelona, 08840 Spain; Departamento de Química Física e Inorgánica, Universitat Rovira i Virgili and Centro de Tecnología Química de Catalunya, Carrer de Marcel•lí Domingo s/n, 43007 Tarragona, Spain; ICREA, Passeig Lluís Companys 23, 08010 Barcelona, Spain

## Abstract

**Background:**

Porous silicon particles (PSiPs) have been used extensively as drug delivery systems, loaded with chemical species for disease treatment. It is well known from silicon producers that silicon is characterized by a low reduction potential, which in the case of PSiPs promotes explosive oxidation reactions with energy yields exceeding that of trinitrotoluene (TNT). The functionalization of the silica layer with sugars prevents its solubilization, while further functionalization with an appropriate antibody enables increased bioaccumulation inside selected cells.

**Results:**

We present here an immunotherapy approach for potential cancer treatment. Our platform comprises the use of engineered silicon particles conjugated with a selective antibody. The conceptual advantage of our system is that after reaction, the particles are degraded into soluble and excretable biocomponents.

**Conclusions:**

In our study, we demonstrate in particular, specific targeting and destruction of cancer cells *in vitro*. The fact that the LD_50_ value of PSiPs-HER-2 for tumor cells was 15-fold lower than the LD_50_ value for control cells demonstrates very high *in vitro* specificity. This is the first important step on a long road towards the design and development of novel chemotherapeutic agents against cancer in general, and breast cancer in particular.

## Background

Cancer is the second cause of death worldwide. In the case of breast cancer, epidemiological studies point to more than one million new cases diagnosed per year and an annual mortality rate close to 450,000 deaths. Particles have shown great potential [1] for drug delivery [2–4] and cancer treatment [5–8]. In most approaches, particles are directed to target cells by antibodies attached to their surfaces, which in the case of in vivo administration, supplements passive targeting through the EPR (enhanced permeability and retention) effect [9]. Some strategies involve heating particles with an external oscillating magnetic or electromagnetic field and causing apoptosis of the nearby cells through magnetothermia [10,11] or photothermia [6,12–15]. These materials suffer certain limitations, such as the large and expensive facilities (i.e. magnetic resonance imaging, MRI) necessary for magnetothermia, and the limited penetration depth of light in the body in the case of photothermia. Other approaches make use of antibody functionalized particles loaded with cancer drugs to deliver the drug to tumor cells [5,16–19]. Porous silicon particles (PSiPs) have been considered a very promising platform for cancer therapy because of their excellent biocompatibility [20] and biodegradability [5,21–23]. In all studies reported to date, PSiPs work either as a passive carrier of an anticancer cargo [5,16–19] or as an element activated by an appropriate trigger, namely light and acoustic waves for particle thermalization [14,24,25] or singlet oxygen generation in photodynamic therapies [8]. Here, we demonstrate that PSiPs themselves can be used as a drug for cancer treatment and how to modulate their activity by taking advantage of their surface functionalization and the enzymatic machinery of eukaryotic cells.

Silicon is characterized by a reduction potential [26] of −1.697 eV to yield silicates or −0.91 eV to yield silica, which is ultimately dissolved as silicates in the presence of water. The low reduction potential makes the reactions violent and even explosive [27] in nanoscaled porous particles [28]. On the other hand, the high tendency of silicon to undergo oxidation is modulated by the spontaneous generation of a passivation layer of SiOx when exposed to open atmosphere. Notably, this passivation layer dissolves in water and particularly in slightly acidic media. The kinetics of the dissolution of this layer can be modulated by surface functionalization of the silica. Thus, by coating with a compact monolayer of an organic molecule, dissolution can be retarded or even prevented.

The processes for obtaining PSiPs have been well known for 20 years [29]. They are mainly based on wet chemistry methods, where the starting material, bulk silicon, is converted into porous silicon, by an electrochemical or stain-etching reaction, followed by a process such as ultrasonication, to break the porous layer into small particles [30,31]. Other, less studied methods, use a bottom-up approach based on chemical vapor deposition techniques, where the starting material is a precursor gas, namely silane or di-silane, which can be decomposed at high temperatures [26]. Under controlled conditions of pressure, temperature and time, such gases nucleate and nanometric porous silicon particles can be obtained [32]. This is a complex process, and the involved mechanisms are still under study (see Methods) [33,34]. In some manner PSiPs can be regarded as a macromolecule resulting from the polymerization of disilane with a dramatic capability of oxidation.

Herein we demonstrate that silicon particles coated with a native silica layer can be engineered with the bio-organic appropriate ligands to target and accumulate into tumour cells. Once inside the cell, the particles are driven to the lysosome were the enzymatic machinery of the cell metabolize the ligands. Then, the exposition of the soluble silica coating to the aqueous lysosomal solution degrades this layer allowing water to react violently with the silicon. As result the target cells dye while the particles are degraded into soluble and excretable biocomponents.

## Results and discussion

**Figure 1:**
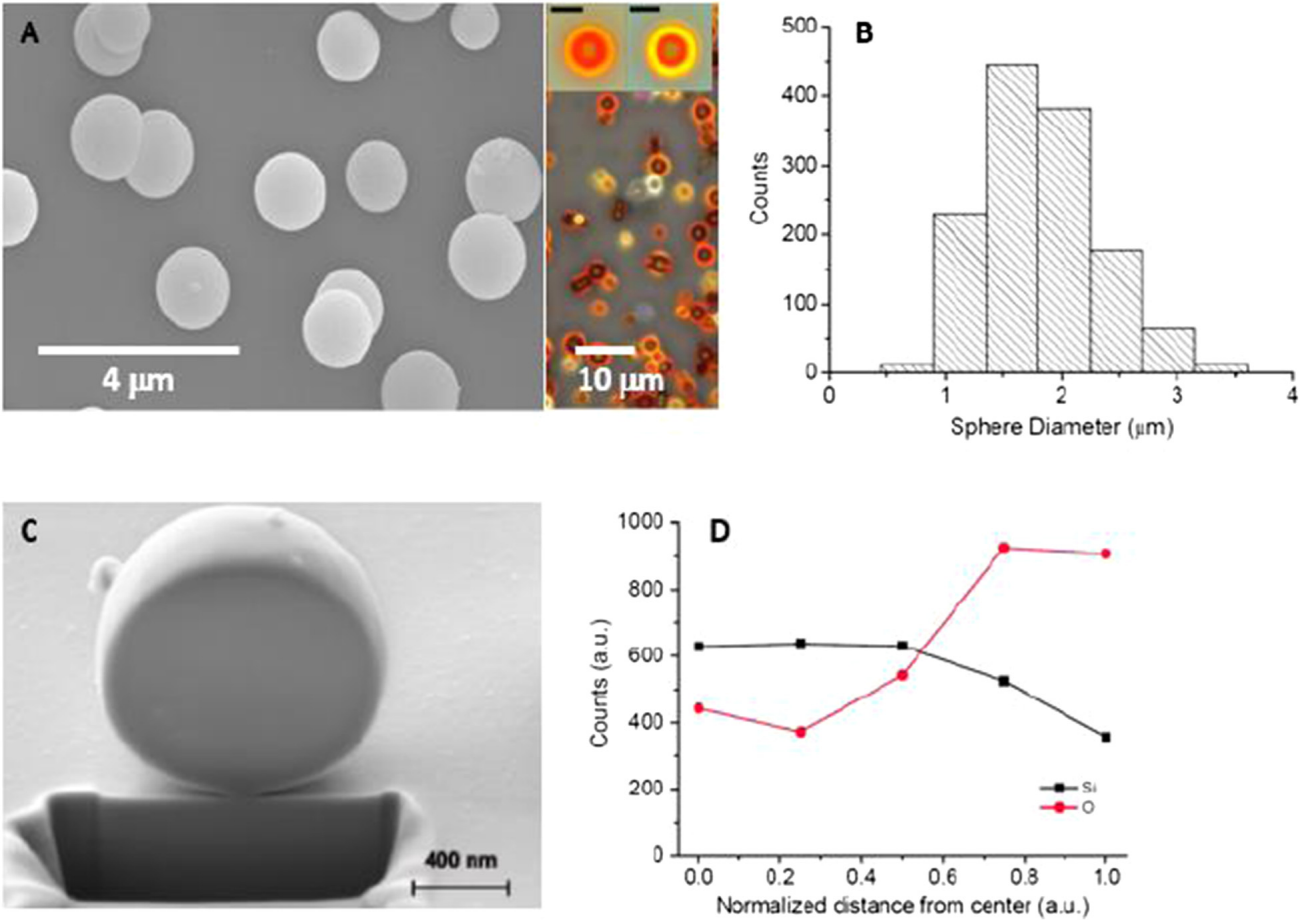
**Silicon particle characterization. (A)** SEM and optical microscopy images of the porous silicon particles as prepared and **(B)** their size distribution. All scale bars in the insets correspond to 2 μm. **(C)** SEM image of a porous silicon colloid carved using the focused ion beam (FIB) technique. **(D)** EDX analysis of materials inside a silicon colloid as a function of the distance from the sphere center, normalized to the sphere radius (d = distance from the sphere center/sphere radius). The graph shows the counts for the Si and O peaks of the EDX spectra at five different points of analysis. The points were selected along a line travelling from the nucleus (d = 0) to the sphere surface (d = 1).

**Figure 2:**
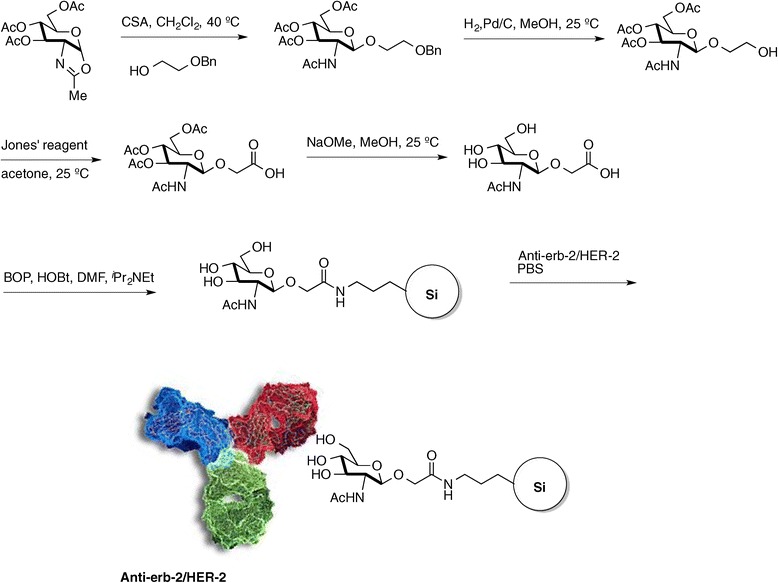
**Synthesis and derivatization of the glucopyranose derivative, and the antibody coupling to the particles.**

Our process to obtain the particles used in this work stem from these methods [35]. The nanostructured particles were highly spherical with a diameter between 1.5 and 2 μm, with an intrinsic photoluminescence in between the dark red and the near infrared (Figure 1A and B) [36]. As prepared, and after extracting the materials from the reactor, exposure to atmospheric oxygen generated a thin layer of silicon oxide (Figure 2C and D). This coating acted as a protective shell and generated an easily functionalizable surface to couple an organic layer would protect the silica from dissolution in physiological media. The enzymatic machinery present in the cell can easily degrade this protective organic shell, which would consequently trigger oxidation reactions of the particles after their internalization by cells. To prove this concept, we used a glucopyranoside derivative that can be easily metabolized by the lysosomal enzyme α-*D*-*glucoside* glucohydrolase. The lysosome has been shown to be a target organelle for most nano- and microparticles [37,38] and thus it can be predicted that after internalization by cells, the particles would be exposed to α-*D*-*glucoside* glucohydrolase.

The sugar and the particle cannot be directly linked without previous modifications. Thus, PSiPs were capped with aminopropylsilane (APS) [39], while the glucopyranoside selected was 2-acetamido-2-deoxy-β-D-glucopyranosyloxyacetic acid (Figure 2, see Methods and SI for details). The benzotriazol-1-yloxytris(dimethylamino)phosphonium hexafluorophosphate/anhydrous 1-hydroxybenzotriazole (BOP/HOBt) coupling method was chosen to generate a peptide bond between the amine-functionalized particle surface and the carboxylic acid group attached to the carbohydrate. Notably, within this configuration, hybrid particles will resist oxidation in physiological media but should be degraded unselectively within any cell, following endocytosis and subsequent localization within the lysosome. Therefore, for anticancer therapy, a selective antibody that targets the surface receptor of the desired cell is necessary.

Consequently, the third step in the preparation of our immunotherapeutic material involved the coupling of a directing vector. HER-2-positive breast cancer is characterized by the amplification of this gene and high expression and activity of its protein [40]. In fact, there is a strong association between HER-2 (ErbB2) tyrosine kinase expression and the aggressiveness and prognosis of the disease. Fortunately, HER-2 amplification confers a selective target for specific treatment and several drugs targeting his receptor are actually used for breast cancer treatment in medical practice, including small molecule tyrosine kinase inhibitors (TKIs) such as gefitinib, erlotinib or lapatinib or monoclonal antibodies such as trastuzumab, pertuzumab or cetuximab [41]. HER-2 is also a standard receptor for specific targeting [42]. For our materials, rather than using standard carbodiimide chemistry, the HER-2 antibody was linked to the particles by taking advantage of its affinity for the sugar. Thus, one of the four glycosylation immunogenic regions was spontaneously coupled to the sugars present on the particle surface, allowing the other three to interact with the cell membrane receptors [43].

**Figure 3:**
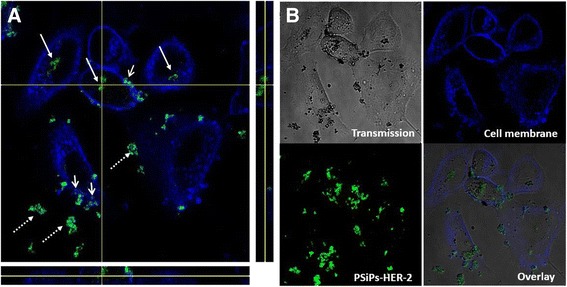
**Particle localization into SK-BR-3 cell lines.** SK-BR-3 (cell membrane labeled in blue) treated with PSiP-HER-2 particles (presented in green) for 24 h, showing the particles inside (arrow) and, outside (dashed arrow) the cells and at the cell membrane (opened arrow). **(A)** Orthogonal view in the 3 planes (X/Y, X/Z and Y/Z) of the particles pointed at the intersection of the X and Y axeis. As seen from all planes, the particles are surrounded by the cell membrane. **(B)** Confocal images of the cells in the transmission channel, the cell membrane and PSiP-HER-2 in fluorescent channels, and overlay of all channels. PSiP-HER-2 were labeled with DyLight 488.

First, to test the interaction of our particles with the cells, the PSiP-HER-2 were internalized by the HER-2 positive cell line, SK-BR-3. The particles could be visualized, surrounded by the cell membrane (Figure 3A). Some particles remained in the extracellular space, while other particles were seen attached to the cell membrane. Despite their micro-size and the degree of agglomeration, the particles could be internalized by SK-BR-3 cells without affecting their viability at an early stage. This is in agreement with other work [37] showing that particles at the micro-scale can be safely incorporated by eukaryotic cells.

**Figure 4:**
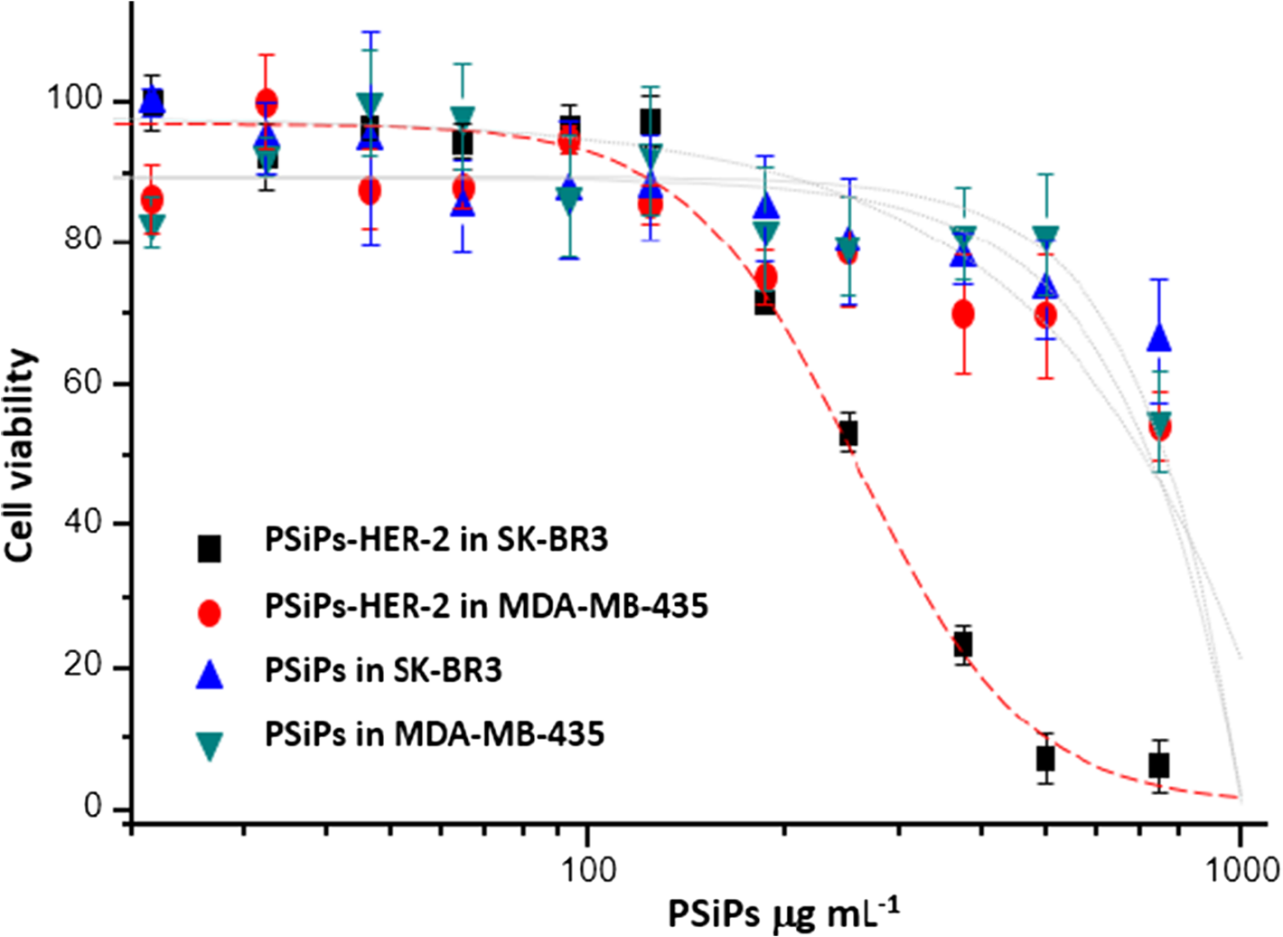
**Cell viability.** Relative cell viability after incubation of SK-BR-3 and MDA-MB-435 cells with PSiPs and PSiPs-HER-2 for 48 h.

Next, we tested the efficiency of the PSiPs functionalized with antibodies against HER-2 receptors (PSiPs-HER-2) for their potential to selectively kill only cells overexpressing HER-2. To this end, we used two different cell lines, one overexpressing the HER-2 receptor (SK-BR-3) and one with its normal expression level (MDA-MB-435) [44]. The cells were seeded in 96-well plates and incubated with different quantities of PSiPs-HER-2 for 48 h, before a resazurin-based viability assay was performed. Resazurin is a nonfluorescent molecule that is reduced by metabolic active cells to the fluorescent resorufin. Thus, the number of viable cells can be determined by measuring resorufin fluorescence. Notably, when SK-BR-3 cells (cells overexpressing the HER-2 receptors) were treated with PSiPs-HER-2, their viability was clearly compromised (Figure 4, black points). On the other hand SK-BR-3 cells treated with PSiPs (Figure 4, blue points) or MDA-MB-435 cells treated with PSiPs or PSiPs-HER-2 (Figure 4, red and green points, respectively) showed a more delayed toxicological response. The LD_50_ (lethal dose killing 50% of the cell population) values are presented in Table 1. Only around 250 μg/mL PSiPs-HER-2 were needed to kill 50% of the SK-BR-3 cells, whereas much higher quantities of the same particles were required under the same conditions to kill the same proportion of MDA-MB-435 cells. On the other hand, PSiPs without attached HER-2 antibodies showed a much lower toxicological response, as their LD_50_ values were very high for both cell types.

**Table 1:** **Calculated LD50 obtained from the dose–response curves shown in Figure** 4

**Cell line**	**Type of NP**	**LD**_**50**_ **(μg/mL)**	**LD**_**50**_ **SD**
SK-BR-3	PSiPs-HER-2	249	10
MDA-MB-435	PSiPs-HER-2	3776	180
SK-BR-3	PSiPs	5603	360
MDA-MB-435	PSiPs	2672	214

These results confirm the efficacy of PSiPs-HER-2 to recognize the HER-2 receptors present on the surface of SK-BR-3 cells to a higher extent than that on MDA-MB-435 cells and to effectively promote their local accumulation. The degree of targeting was more than sufficient to accelerate the death of the targeted cancer cells.

## Conclusions

In summary, we present here an immunotherapy approach for potential cancer treatment. Our platform comprises the use of engineered silicon particles conjugated with a selective antibody. The conceptual advantage of our system is that after reaction, the particles are degraded into soluble and excretable biocomponents. In our study, we demonstrate in particular, specific targeting and destruction of cancer cells *in vitro*. The fact that the LD_50_ value of PSiPs-HER-2 for tumor cells was 15-fold lower than the LD_50_ value for control cells demonstrates very high *in vitro* specificity. This is the first important step on a long road towards the design and development of novel chemotherapeutic agents against cancer in general, and breast cancer in particular.

## Methods

### Synthesis of porous silicon microspheres

Our method for producing porous silicon microspheres is based on the decomposition of disilane gas (Si_2_H_6_) by chemical vapor deposition (CVD). This is similar to the synthesis of silicon colloids [45], where the gas is introduced in a reactor whose walls are heated at high temperatures for a certain time, usually higher than 400°C. During this procedure, Si_n_H_m_ clusters grow in the gas phase [46] and these become highly spherical, micrometer-sized particles, through surface tension forces. At the same time, the process of hydrogen desorption from the clusters progressively reduces the hydrogen content until they become hydrogenated amorphous silicon (a:Si-H) colloids. To obtain porous silicon microspheres, the heating process is stopped at an early stage, before the formation of amorphous silicon colloids is complete. In this way, porous particles with an undetermined composition of silicon and hydrogen atoms are obtained.

Porous silicon particles for this work were synthesized using a di-silane decomposition temperature of 400°C. The absolute gas pressure in the reactor was about 0.25 atm at room temperature and we used decomposition times from 1 to 2 minutes, measured from the stage where the gas reaches the target temperature.

Silicon and oxygen content of porous silicon colloids were analyzed by electron dispersive X-ray (EDX) measurements. For this purpose, colloids were carved using a focus ion beam (FIB) technique (FEI Helios NanoLab Omniprobe). Figure 1C shows a scanning electron microscope (SEM) image of a carved colloid, 1.2 μm in diameter. The carved surface appears to be flat because the ion gun of the FIB removes material and deposits it at the same time on the surface of the particle, thus hiding porous structures. Nevertheless, because the EDX interaction volume penetrates the region where pores are less modified by the FIB carving action, one can extract useful information about the cavity structure. Figure 1D shows the EDX analysis corresponding to silicon and oxygen in different regions of the particle, from the cavity center to the cavity surface. This shows two key results: a) the oxygen content appears to be completely within the cavity and b) the oxygen (silicon) concentration increases (decreases) from the center to the surface. This result supports the hypothesis that porous silicon colloids possess a gradient, or onion-like, porous structure, as images taken by optical microscopy suggest. In addition, it indicates that porosity is higher at the surface of the colloid than within it.

### Synthesis of the 2-acetamido-2-deoxy-β-d-glucopyranosyloxyacetic acid

Solvents were dried according to published methods and distilled before use. All other reagents were commercial compounds of the highest purity available. Unless otherwise indicated, all reactions involving air- and moisture-sensitive materials were carried out under an argon atmosphere, while those not involving aqueous reagents were carried out in oven-dried glassware. Analytical thin layer chromatography (TLC) was performed on aluminum plates with Merck Kieselgel 60 F254 and visualized by UV irradiation (254 nm) or by staining with an ethanolic solution of phosphomolybdic acid. Flash column chromatography was carried out using Merck Kieselgel 60 (230–400 mesh) under pressure. ^1^H NMR spectra were recorded in CDCl_3_ and D_2_O, at ambient temperature on an AMX-400 spectrometer at 400 MHz, with residual protic solvent as the internal reference [CDCl_3_, δ_H_ = 7.26 ppm]; chemical shifts (δ) are given in parts per million, and coupling constants (*J*) are given in Hertz. The proton spectra are reported as follows: δ (multiplicity, coupling constant *J*, number of protons, assignment).



2-(Benzyloxy)ethanol 2. To a mixture of NaH (3.4 g, 0.085 mol, 60% w/w in mineral oil) in tetrahydrofuran (THF, 150 mL), ethylene glycol 1 (25.1 mL, 0.45 mol) was added and the mixture was stirred for 1 h at 25°C. Then, benzyl bromide (8.9 g, 0.075 mol) was added and the reaction was refluxed for 12 h. After cooling the mixture (0°C), a saturated aqueous solution of NH_4_Cl was added, the solvent was evaporated and the mixture was extracted with EtOAc (3×). The combined organic layers were washed with a saturated aqueous solution of NH_4_Cl and brine, and dried (Na_2_SO_4_). The solvent was evaporated to afford 11.22 g (98% yield) of a colorless oil identified as 2-(benzyloxy)ethanol 2. The spectroscopic data were identical to those described in the literature [47]. ^1^H-NMR (400 MHz, CDCl_3_): δ 7.40-7.30 (m, 5H), 4.58 (s, 2H), 3.78 (t, *J* = 5.0 Hz, 2H), 3.61 (t, *J* = 4.9 Hz, 2H), 2.06 (br, 1H, OH) ppm.



1,3,4,6-tetra-*O*-acetyl-α/β-*N*-acetylglucosamine 4. *N*-acetyl-D-glucosamine 3 (5 g, 22.6 mmol) was dissolved in pyridine (36 mL) and acetic anhydride (25 mL) was added dropwise at 0°C. The reaction mixture was stirred at 25°C for 24 h, then diluted with CH_2_Cl_2_ and washed consecutively with cold water, a saturated aqueous solution of NaHCO_3_, and a 10% aqueous solution of CuSO_4_. The organic layer was dried (Na_2_SO_4_) and the solvent was evaporated to obtain 6.60 g (75%) of a white solid identified as 1,3,4,6-tetra-*O*-acetyl-α/β-*N*-acetylglucosamine 4. The spectroscopic data were identical to those described in the literature [48]. ^1^H-NMR (400 MHz, CDCl_3_): δ 6.17 (d, *J* = 3.6 Hz, 1H), 5.64 (d, *J* = 9.3 Hz, 1H), 5.30-5.20 (m, 2H), 4.49 (ddd, *J* = 10.6, 8.9, 3.6 Hz, 1H), 4.25 (dd, *J* = 12.5, 4.1 Hz, 1H), 4.07 (dd, *J* = 12.5, 2.4 Hz, 1H), 4.00 (ddd, *J* = 9.6, 4.0, 2.3 Hz, 1H), 2.20 (s, 3H), 2.09 (s, 3H), 2.06 (s, 3H), 2.05 (s, 3H), 1.94 (s, 3H) ppm.



4′,5′-Dihydro-2′-methyloxazolo [5′,4′:1,2]-3,4,6-tri-*O*-acetyl-1,2-dideoxy-α-D-glucopyranoside 5. To a solution of 1,3,4,6-tetra-*O*-acetyl-α/β-*N*-acetylglucosamine 4 (2.49 g, 6.4 mmol) in dichloroethane (25 mL) TMSOTf (1.8 mL, 9.6 mmol) was added and the reaction was stirred for 2 h at 55°C and for 12 h at 25°C. A saturated aqueous solution of NaHCO_3_ was added and the mixture was extracted with CH_2_Cl_2_ (3×). The combined organic layers were washed with a saturated aqueous solution of NaHCO_3_ and dried (Na_2_SO_4_) and the solvent was evaporated. The resulting residue was purified by column chromatography (silica gel, 97:3 CH_2_Cl_2_/MeOH) to afford 1.86 g (78%) of 4′,5′-dihydro-2′-methyloxazolo [5′,4′:1,2]-3,4,6-tri-*O*-acetyl-1,2-dideoxy-α-D-glucopyranoside 5. The spectroscopic data were identical to those described in the literature [49]. ^1^H-NMR (400 MHz, CDCl_3_): δ 5.98 (d, *J* = 7.4 Hz, 1H), 5.27 (t, *J* = 2.4 Hz, 1H), 4.94 (ddd, J = 9.2, 2.0, 1.2 Hz, 1H), 4.19-4.14 (m, 3H), 3.62 (dt, *J* = 8.8, 4.3 Hz, 1H), 2.12 Hz (s, 3H), 2.11 (s, 3H), 2.10 (s, 3H), 2.09 (s, 3H) ppm.



2-Hydroxyethyl-2-acetamido-3,4,6-tri-O-acetyl-2-deoxy-β-D-glucopyranoside 7. 10-(*R*)-camphorsulfonic acid (1.40 g, 5.02 mmol) and 2-(benzyloxy)ethanol 2 (7.63 g, 50.2 mmol) were added to a stirred solution of 4′, 5′-dihydro-2′-methyloxazolo [5′,4′:1,2]-3,4,6-tri-*O*-acetyl-1,2-dideoxy-α-D-glucopyranoside 5 (1.86 g, 5.02 mmol) and powdered 4 Å molecular sieves (ca. 8 g) in CH_2_Cl_2_ (30 mL) and the reaction was stirred at 40°C for 14 h. The mixture was cooled to 0°C and a saturated aqueous solution of NaHCO_3_ (60 mL) and CH_2_Cl_2_ (30 mL) was added. The layers were separated and the organic layer was washed with a saturated aqueous solution of NaHCO_3_, brine and dried (Na_2_SO_4_), and the solvent then evaporated. The residue was purified by column chromatography (hexane/EtOAc 50:50 to CH_2_Cl_2_/MeOH 95:5) providing 2-*O*-benzyloxyethyl-2-acetamido-3,4,6-tri-O-acetyl-2-deoxy-β-D-glucopyranoside 6 as a colorless solid. The spectroscopic data of the product were identical to those described in the literature [50]. ^1^H-NMR (400 MHz, CDCl_3_): δ 7.4-7.3 (m, 5H), 5.50 (d, *J* = 8.8 Hz, 1H), 5.25 (dd, *J* = 10.4, 9.4 Hz, 1H), 5.09 (t, *J* = 9.6 Hz, 1H), 4.76 (d, *J* = 8.4 Hz, 1H), 4.56 (app s, 2H), 4.27 (dd, *J* = 12.3, 4.7 Hz, 1H), 4.20-4.15 (m, 2H), 3.99 (dt, *J* = 11.5, 3.9 Hz, 1H), 3.80 (ddd, *J* = 18.2, 14.7, 11.2 Hz, 1H), 3.70-3.65 (m, 3H), 2.09 (s, 3H), 2.06 (s, 3H), 2.03 (s, 3H), 1.87 (s, 3H) ppm.

A mixture of 2-*O*-benzyloxyethyl-2-acetamido-3,4,6-tri-*O*-acetyl-2-deoxy-β-D-glucopyranoside 6 (0.19 g, 0.36 mmol) and Pd/C (10%, 0.02 g) in MeOH (3 mL) was stirred under an H_2_ atmosphere for 7 h at 25°C. The reaction was filtered through Celite and the solvent was evaporated to afford 0.15 g (96%) of a solid identified as 2-hydroxyethyl-2-acetamido-3,4,6-tri-*O*-acetyl-2-deoxy-β-D-glucopyranoside 7. The spectroscopic data of the product were identical to those described in the literature [50]. ^1^H-NMR (400 MHz, CDCl_3_): δ 5.58 (d, *J* = 9.0 Hz, 1H), 5.25 (dd, *J* = 10.6, 9.4 Hz, 1H), 5.07 (t, *J* = 9.6 Hz, 1H), 4.71 (d, *J* = 8.3 Hz, 1H), 4.2-4.10 (m, 2H), 3.9-3.8 (m, 2H), 3.7-3.6 (m, 2H), 2.58 (br, 1H, OH), 2.11 (s, 3H), 2.06 (s, 3H), 2.05 (s, 3H), 1.98 (s, 3H) ppm.



2-Acetamido-3,4,6-tri-*O*-acetyl-2-deoxy-β-D-glucopyranosyloxyacetic acid 8. Jones reagent (3.5 M, 0.2 mL, 0.69 mmol) was added to a stirred solution of 2-hydroxyethyl-2-acetamido-3,4,6-tri-*O*-acetyl-2-deoxy-β-D-glucopyranoside 7 (0.15 g, 0.35 mmol) in acetone (2 mL) at 0°C and the reaction was stirred for 13 h at 25°C. Isopropanol was added and the solvent was evaporated. After the addition of CH_2_Cl_2_ (10 mL) and brine (10 mL), the layers were separated, the organic layer was washed with brine and dried (Na_2_SO_4_), and the solvent was evaporated to afford 0.11 g (73%) of a white solid that was identified as 2-acetamido-3,4,6-tri-*O*-acetyl-2-deoxy-β-D-glucopyranosyloxyacetic acid 8 The spectroscopic data of the product were identical to those described in the literature [50]. ^1^H-NMR (400 MHz, CDCl_3_): δ 6.57 (br s, 1H), 5.22 (t, *J* = 9.6 Hz, 1H), 5.11 (t, *J* = 9.6 Hz, 1H), 4.75 (d, *J* = 8.1 Hz, 1H), 4.35 (app s, 2H), 4.28 (dd, *J* = 12.1, 4.9 Hz, 1H), 4.15 (dd, *J* = 10.7, 2.1 Hz, 1H), 4.1-4.0 (m, 1H), 3.7-3.6 (m, 1H), 2.11 (s, 3H), 2.06 (s, 3H), 2.03 (s, 3H), 1.97 (s, 3H) ppm.



2-Acetamido-2-deoxy-β-D-glucopyranosyloxyacetic Acid 9. Sodium methoxide (0.02 g, 0.332 mmol) was added to a stirred solution of 2-acetamido-3,4,6-tri-*O*-acetyl-2-deoxy-β-D-glucopyranosyloxyacetic acid 8 (0.114 g, 0.255 mmol) in MeOH (6 mL) and the reaction mixture was stirred at 25°C for 24 h. After the addition of Dowex-200 the mixture was filtered and the solvent was evaporated. The residue was dissolved in H_2_O and dried under vacuum to afford 0.071 g (99%) of a solid identified as 2-acetamido-2-deoxy- β -D-glucopyranosyloxyacetic acid 9. The spectroscopic data of the product were identical to those described in the literature [50]. ^1^H-NMR (400 MHz, D_2_O): δ 4.49 (d, *J* = 8.4 Hz, 1H), 4.18 (s, 2H), 3.83 (d, *J* = 12.2 Hz, 1H), 3.70-3.60 (m, 2H), 3.50-3.40 (m, 1H), 3.40-3.30 (m, 2H), 1.97 (s, 3H) ppm.

### Particle functionalization

A suspension of aminopropylsilane (APS)-coated silicon particles was produced by treatment of 0.1 g silica particles with APS (20 μL) in 2-propanol (5 mL) at 80°C for 2 h. The particles were centrifuged at 3800 rpm for 30 min to remove the excess APS, followed by replacement of the supernatant solution by isopropanol. The particles were re-dispersed by shaking (ultrasound) for 10 min. This protocol was repeated two more times [51].

The particles were centrifuged at 3800 rpm for 30 min and the supernatant was replaced by dimethylformamine (DMF). Then, a solution of 2-acetamido-2-deoxy-β-D-glucopyranosyloxyacetic acid 9 (14 mg), benzotriazol-1-yloxy)tris(dimethylamino)phosphonium hexafluorophosphate (BOP, 29 mg), hydroxybenzotriazole (HOBt, 7 mg) in DMF (3 mL) and diisopropylethylamine (30 μL) were added and the suspension was stirred for 12 h at 25°C. The particles were centrifuged at 3800 rpm for 30 min, washed with DMF and the supernatant then replaced by H_2_O. The particles were re-dispersed by shaking (ultrasound) for 10 min. The supernatant was replaced by phosphate buffered saline (PBS, 1×).

To a suspension of the particles in PBS (2 mL) 250 μL of a solution of anti-erbB-2/HER-2 (1 μL/L in PBS) was added and the mixture was shaken at 0°C for 12 h. The particles were centrifuged at 3800 rpm for 10 min, and the supernatant was replaced with PBS. The particles were re-dispersed by shaking (ultrasound) for 3 min at 0°C.

### Cell culture and viability assay

Materials: Dulbecco’s modified eagle’s medium (DMEM, #30-2002) and McCoy’s 5a medium (#30-2007) were purchased from ATCC. Fetal bovine serum (FBS, #S0615) was obtained from Biochrom AG and penicillin/streptomycin (#15140-122) from Gibco (#15140-122). L-glutamine (#25030-024) was purchased from Life Technologies. The viability assay based on Resazurin (#TOX8) and the 96-well plates (#CLS3603) in which the viability assay was carried out were obtained from Sigma-Aldrich (St Louis, MO, USA).

MDA-MB-435 human epithelial cells (ATCC #HTB-129) were seeded and grown in growth medium (DMEM with 4.5 g/L glucose supplemented with 10% FBS, 1% L-glutamine (200 mM) and 1% penicillin/streptomycin. SK-BR-3 human breast adenocarcinoma cells (ATCC #HTB-30) were seeded and grown in another growth medium (McCoy’s medium supplemented with 10% FBS, 1% L-glutamine (200 mM) and 1% penicillin/streptomycin.

Imaging of the particle internalization: SK-BR-3 cells were seeded on an Ibidi 8-well plate (2E4 cells/well) and left to adhere and grow for 4 days. Subsequently, the cells were incubated with 30 μg/mL PSiP-HER-2 particles for 24 h. The cells were then washed intensively and the cell membrane stained with 20 μg/mL cell mask deep red for 10 min at 37°C before imaging. Images of living cells using transmitted and reflected light were taken with a confocal laser scanning microscope (NIKON TE2000-E). A 650 LP filter was used to collect the fluorescence signal of the cell membrane after excitation at 633 nm, whereas a 515/30 BP filter was used to collect the light reflected by the DyLight 488 labeled PSiP-HER-2 after irradiation with a 488 nm laser line. As previously reported, the particles exhibit intrinsic photoluminescence because of the presence of the microcavities [35,36].

Viability assay: 20,000 cells (MDA-MB-435 or SK-BR-3) were seeded per well in a 96-well plate and incubated in 100 μL of the corresponding cell medium for 48 h at 37°C and 5% CO_2_. After this time, different concentrations of particles (PSiNPs or PSiNPs-HER-2) were added and cells were incubated for another 48 h. The viability assay was repeated three times at each concentration. Control viability assays were performed using cells without particles and particles without cells. After incubation, cellular viability was probed. Cells were washed with PBS and a solution of 10% of resazurin in growth medium was added to each well. Cells were placed in the incubator for 3 h (37°C and 5% CO_2_). Resazurin is a nonfluorescent molecule that is reduced from the oxidized to the reduced form, resofurin, by metabolically active cells. Resofurin is fluorescent, has a maximum emission wavelength at 585 nm (red emission), and can be excited from 530 to 560 nm. The fluorescent emission intensity originating from resofurin is proportional to the number of metabolically active (=living) cells. Fluorescence emission was measured with a Fluorolog-3 spectrofluorometer equipped with a microwell plate reader (MicroMax 384) from Horiba JOBIN YVON. The samples were excited at 560 nm and the emission spectra were collected from 572 to 650 nm. Background was subtracted from the spectra. As the position of the maximum emission, wavelength can be slightly shifted, so the peak emission was averaged from 584 to 586 nm. The emission peak intensity values were normalized, considering a cell viability of 100% for the control experiments, in which no particles had been added to the cells. The normalized fluorescence emission peak intensities were plotted against the logarithm of the particle concentration, *cf*. Figure 4. Dose–response curves were obtained for SK-BR-3 and MDA-MB-435 exposed to different concentrations of Si-NPs and SiNPs-HER2, *cf*. Figure 4. The results were fitted to sigmoidal curves and the inflexion point was calculated. The inflexion point represents the LD_50_ value, which in this case is the concentration of particles at which cell viability is reduced to 50%, i.e. 50% of the cells are no longer metabolically active. The calculated LD_50_ values are shown in Table 1.
